# Beta Cell Dysfunction and Insulin Resistance

**DOI:** 10.3389/fendo.2013.00037

**Published:** 2013-03-27

**Authors:** Marlon E. Cerf

**Affiliations:** ^1^Diabetes Discovery Platform, South African Medical Research CouncilCape Town, South Africa

**Keywords:** beta cell compensation, diabetes, obesity, oxidative stress, proliferation

## Abstract

Beta cell dysfunction and insulin resistance are inherently complex with their interrelation for triggering the pathogenesis of diabetes also somewhat undefined. Both pathogenic states induce hyperglycemia and therefore increase insulin demand. Beta cell dysfunction results from inadequate glucose sensing to stimulate insulin secretion therefore elevated glucose concentrations prevail. Persistently elevated glucose concentrations above the physiological range result in the manifestation of hyperglycemia. With systemic insulin resistance, insulin signaling within glucose recipient tissues is defective therefore hyperglycemia perseveres. Beta cell dysfunction supersedes insulin resistance in inducing diabetes. Both pathological states influence each other and presumably synergistically exacerbate diabetes. Preserving beta cell function and insulin signaling in beta cells and insulin signaling in the glucose recipient tissues will maintain glucose homeostasis.

## Introduction

Both beta cell dysfunction and insulin resistance lead to persistent hyperglycemia which characterizes type 2 diabetes. Many of the susceptibility genes associated with type 2 diabetes by genome-wide investigations (GWAS) were identified as regulators of cell turnover or regeneration (McCarthy and Hattersley, 2008). Most risk variants for type 2 diabetes in healthy populations act through impairing insulin secretion (resulting in beta cell dysfunction) rather than insulin action (resulting in insulin resistance) which establishes that inherited abnormalities of beta cell function or mass (or both) are critical precursors in type 2 diabetes (Florez, 2008; McCarthy, 2010; Voight et al., 2010; Petrie et al., 2011). Recent linkage studies and GWAS have identified >40 genes that increase the risk of type 2 diabetes with the most important diabetes susceptibility gene identified as transcription factor 7-like 2 (*tcf7l2*), which increases diabetes risk 1.7-fold (Ashcroft and Rorsman, 2012). Potassium voltage-gated channel, KQT-like subfamily, member 1 (*Kcnq1*) is a type 2 diabetes susceptibility gene implicated in reduced beta cell function and decreased insulin secretion (Bonnefond et al., 2010). Common variants in several neonatal diabetes mellitus and maturity-onset diabetes of the young (MODY) [e.g., potassium inwardly rectifying channel, subfamily J, member 11 (*kcnj11*), glucokinase (*gck*), hepatocyte nuclear factor 4 alpha (*hnf1*α), and *hnf1*β] are recognized as type 2 diabetes susceptibility variants 6 and 43 (Bonnefond et al., 2010). Reduced expression of the transcription factor, prospero homeobox 1 (Prox1), by cis-regulatory variants altered beta cell insulin secretion which conferred susceptibility to type 2 diabetes (Lecompte et al., 2013).

**Figure 1:**
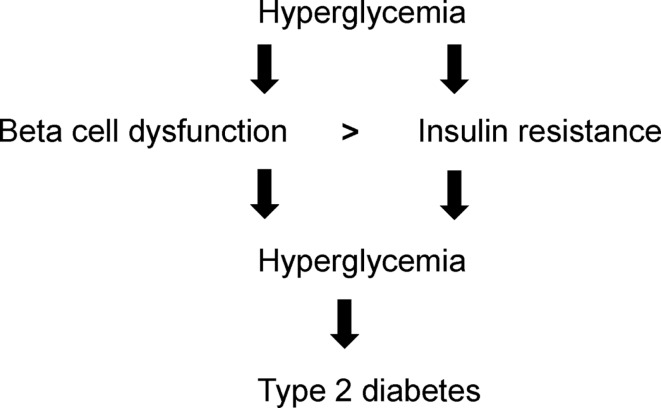
**Hyperglycemia-induced beta cell dysfunction, insulin resistance, and type 2 diabetes**.

Beta cell dysfunction is the critical determinant for type 2 diabetes (Ashcroft and Rorsman, 2012) which is compounded by insulin resistance. The interplay between beta cell dysfunction and insulin resistance remains highly complex. The onset of hyperglycemia can trigger both beta cell dysfunction and insulin resistance (Figure 1). Beta cell dysfunction is more severe than insulin resistance (Figure 1). With beta cell dysfunction, insulin secretion is impaired whereas with insulin resistance, insulin may still be secreted but insulin insensitivity manifests in target tissues. As beta cell dysfunction and insulin resistance exacerbate, hyperglycemia amplifies leading to the progression to type 2 diabetes (Figure 1). This review focuses on beta cells: physiology and integrity, demise and dysfunction, compensation, and preservation.

## Beta Cell Physiology and Integrity

Adequate and proper beta cell function requires normal beta cell integrity which is critical for the appropriate response to perpetual fluctuating metabolic demand for insulin. Genes implicated in cell-cycle regulation are suggested to influence beta cell mass during development (Ashcroft and Rorsman, 2012). A decrease in beta cell mass of ≤60% has been reported in type 2 diabetes (Butler et al., 2003), which parallels the extent of reduction in glucose-stimulated insulin secretion (GSIS) (Del Guerra et al., 2005) but, however, considerably lower decrements have been found (Rahier et al., 2008). Although beta cell mass plays a role in type 2 diabetes, beta cell function rather than number is more critical in the etiology of type 2 diabetes (Ashcroft and Rorsman, 2012). Beta cells are resilient and will compensate to cope with insulin demand despite reduced numbers.

Under physiological conditions, the maintenance of blood glucose concentrations within a narrow physiological range relies on coordinated regulation of insulin secretion through nutrient availability, hormones, and neural inputs (Schrimpe-Rutledge et al., 2012). Amongst these factors, glucose is by far the most potent and physiologically important regulator of beta cell function through coordinated stimulation of insulin gene transcription, proinsulin biosynthesis, and insulin secretion from beta cells (Henquin et al., 2006). The highly coordinated regulation of gene and protein expression in response to glucose stimulation is responsible for many established cellular functions such as glycolysis and insulin biosynthesis/secretion, but also for unknown responses (Schrimpe-Rutledge et al., 2012). Glucose is a major regulator of transcription and translation in beta cells, an effect that is necessary for the long-term maintenance of the highly differentiated state of the cell and the secretory requirements imposed by prolonged elevations of glucose concentrations (Schuit et al., 2002). In addition, considering that beta cells are highly metabolically active and that insulin secretion is tightly coupled to glucose metabolism, the most highly glucose regulated proteins are implicated in glucose metabolism (Schrimpe-Rutledge et al., 2012). Glucose is therefore a critical determinant of beta cell function – persistent hyperglycemia may exhaust beta cells whereas hypostimulation may prime beta cells for low glycemic states (fasting and starvation) potentially limiting their response to hyperglycemic excursions.

## Beta Cell Demise and Dysfunction

Beta cell insults include cytokine-induced inflammation, obesity and insulin resistance, and overconsumption of saturated fat and free fatty acids (FFA). A progressive decline of beta cell function leading to beta cell exhaustion precedes beta cell demise (Ferrannini, 2010; Talchai et al., 2012). Loss of beta cell mass and function are central to the development of both type 1 and 2 diabetes (Stoffers, 2004).

### Cytokines, oxidative stress, endoplasmic reticulum stress, and inflammation

Specific proinflammatory cytokines induce an inflammatory response. Inflammation is associated with obesity and both are linked to insulin resistance. Proinflammatory cytokines cause beta cell death via the induction of mitochondrial stress and other responses (Cnop et al., 2005; Eizirik and Cnop, 2010; Gurgul-Convey et al., 2011). Cytokines secreted by immune cells that have infiltrated the pancreas are reported to be crucial mediators of beta cell destruction (Lin et al., 2012).

Chronic exposure hyperglycemia, leading to oxidative stress and inflammation, may induce changes in the regulation of gene expression that converge on impaired insulin secretion and increased apoptosis (Gilbert and Liu, 2012). Oxidative stress leads to damage in organelles, particularly the mitochondria and cellular proteins, lipids, and nucleic acids (Simmons, 2007); it also initiates and contributes to both endoplasmic reticulum (ER) stress (Kaneto et al., 2005; Hotamisligil, 2010) and autophagy (Lee et al., 2012), although these mechanisms are not completely defined (Yuzefovych et al., 2013). ER stress is associated with beta cell apoptosis in type 2 diabetes (Marchetti et al., 2007).

Reactive oxygen species (ROS) and reactive nitrogen species (RNS) are formed during both cytokine-mediated proinflammatory beta cell aggression in type 1 diabetes and glucolipotoxicity-mediated beta cell dysfunction in type 2 diabetes (Evans et al., 2003; Cnop et al., 2005; Robertson et al., 2007; Lenzen, 2008). Increased ROS production from mitochondrial overstimulation and RNS from excess nitric oxide (NO) production in beta cells leads to the inhibition of the electron transport chain resulting in reduced energy production, DNA damage, and the formation of advanced glycation end products (AGEs) (Drews et al., 2010).

The limited glycolytic capacity of beta cells can generate ROS and the ensuing oxidative stress can uncouple glucose sensing from insulin secretion (Robertson, 2004). Beta cells are highly dependent on ATP production for GSIS and are vulnerable to excess ROS because of their inherently low expression of antioxidant enzymes (Simmons, 2007). The imbalance or reduced availability of nutrients to beta cells, small repeated increases in ROS production, lower ATP synthesis, and inadequate antioxidant balance may predispose to beta cell dysfunction (Reusens et al., 2011).

### Obesity and insulin resistance

Obesity is a state of low grade inflammation. Adipose tissue is a major source of inflammation with the infiltration of macrophages as the primary source of cytokines in obese individuals (Stienstra et al., 2007). The beta cells secrete insulin to facilitate glucose uptake into glucose recipient organs (mainly the muscle, brain, liver, and adipose tissue). Obesity is a key risk factor for type 2 diabetes as it desensitizes glucose recipient organs to the action of insulin. Saturated fats are strongly associated with insulin resistance and beta cell dysfunction. High saturated fats in circulation, derived mainly from diets or even from lipolysis of fat depots, lead to fatty acids and glucose competing for uptake and metabolism in tissues. With persistent hyperglycemia, increased saturated FFA induce a glucolipotoxic state that is detrimental to beta cells by increasing oxidative stress, subsequently reducing insulin synthesis and secretion thereby compromising both beta cell structure and function.

The genetic architecture of quantitative indices of beta cell function and of insulin resistance differ markedly: given the same individuals, sample sizes, and biochemical measurements, more signals were described for beta cell function compared to insulin resistance (Dupuis et al., 2010; Voight et al., 2010). This infers that beta cell function supersedes insulin resistance as the critical determinant of type 2 diabetes. Further, as obesity is a critical determinant of insulin resistance, adiposity may modulate the genetic determinants of insulin resistance and contribute to the heterogeneity of type 2 diabetes (Manning et al., 2012). In addition, the adipokine hormones and proinflammatory cytokines that are produced by adipose tissue can influence insulin signaling via diverse mechanisms (Shoelson et al., 2006; Trujillo and Scherer, 2006) and these processes may interact with genetic variants influencing insulin resistance pathways (Manning et al., 2012). Adiposity levels have the potential to perturb the physiological milieu in which genetic variants in insulin signaling pathways operate (Manning et al., 2012). Therefore adiposity, and particularly fat in the pancreas and major glucose recipient organs, will exacerbate insulin resistance which consequently impairs beta cell function. Obesity also increases insulin demand therefore hyperfunction of beta cells may exhaust beta cells resulting in beta cell dysfunction.

By upregulating insulin secretion (Bergman, 2005) and the degree to which individuals are capable of achieving this determines whether or not an individual develops diabetes (Ashcroft and Rorsman, 2012). This, in turn, is influenced by their genetic constitution which explains why some obese and insulin resistant individuals do not develop type 2 diabetes; their beta cells can compensate (Ashcroft and Rorsman, 2012). In those individuals who develop diabetes, beta cell compensation declines due to hyperglycemia resulting in a persistent progressively increasing metabolic load that is influenced by factors such as aging and an increase in the number and severity of diabetogenic risk factors.

### Saturated fat and FFA trigger beta cell dysfunction and insulin resistance

Higher body mass indices (BMIs) may potentiate the effect of genetic variants on insulin resistance pathways, an effect that could be attributed to tissue-specific responses to the obesogenic environment (Prudente et al., 2009). The effects of FFA on beta cell function are time dependent. Short-term exposure to FFA increases GSIS which results in increased insulin secretion following a mixed meal and enables storage of excess calories as fat (Ashcroft and Rorsman, 2012) which contributes to overweight and obesity. FFA may account for the compensatory upregulation of beta cell function in response to insulin resistance (Stefanovski et al., 2011). Conversely, long-term exposure to FFA suppresses GSIS and has been suggested to involve impaired glucose metabolism, reduced insulin biosynthesis, and beta cell loss (Yaney and Corkey, 2003; Poitout and Robertson, 2008).

Obesity and high fat feeding mimic the effects of long-term incubation of islets with FFA on insulin secretion and Ca^2+^-channel distribution (Collins et al., 2010) which correlate with an increased amount of fat within the islets (Hoppa et al., 2009) and the surrounding exocrine pancreas (Pinnick et al., 2008). There is also an inverse correlation between the amount of fat in the human pancreas and GSIS, with glucose tolerance and insulin secretion improving in parallel with a reduction in pancreatic fat (Tushuizen et al., 2007). Intra-pancreatic or intra-islet fat depots may therefore provide a long-term local source of FFA that adversely affects beta cell function (Ashcroft and Rorsman, 2012). Obesity coupled to insulin resistance increases the functional demand per beta cell which would increase the burden and accelerate beta cell dysfunction. The pathogenesis of beta cell dysfunction may, to a certain extent, mimic hepatic steatosis: intra-tissue fat depots induce inflammation thereby triggering cellular demise and dysfunction. Intra-islet, in particular intra-beta cell, fat could therefore impair GSIS and insulin signaling in islets. This likely represents a mechanism for beta cell dysfunction and reduced beta cell compensation that impairs GSIS by non-glucose sensing by beta cells similar to impaired insulin signaling and consequently glucose uptake in glucose recipient tissues that triggers insulin resistance.

Islet inflammation (insulitis) in type 2 diabetes is attributed to nutrient overload leading to metabolic exhaustion in beta cells (Donath and Shoelson, 2011). This can lead to localized synthesis of cytokines, resulting in recruitment of immune cells to the site of production (Donath and Shoelson, 2011) which will trigger beta cell dysfunction and exacerbate insulin resistance. Nuclear factor of kappa light polypeptide gene enhancer in B cells (NF-κB) has a central regulatory role in FFA-induced inflammation and beta cell death (Choi et al., 2012). The saturated FFA, palmitate, induces beta cell dysfunction *in vivo* by activating inflammatory processes within mouse islets (Eguchi et al., 2012). Palmitate treatment increased expression of major cytokines implicated in beta cell dysfunction (Choi et al., 2012), viz., interleukin (IL) 6, IL8 (CXCL1), IP10 [chemokine (C-X-C motif) ligand 10 (CXCL10)], MCP1 (CCL2), and MIP1A (CCL3) (Donath et al., 2010), where they may influence beta cells in an autocrine manner (Choi et al., 2012). Insulin sensitivity is impaired by saturated FFA and improved by polyunsaturated FFA (Siri-Tarino et al., 2010). In rats, saturated FFA have shown to increase intramuscular palmitic acid accumulation that may lead to insulin resistance (Reynoso et al., 2003). In humans, a positive association between serum FFA composition and diabetes was reported (Vessby et al., 1994; Coelho et al., 2011).

## Beta Cell Compensation

Upon beta cell demise, beta cell compensation occurs to restore beta cell physiology. Optimal control of blood glucose concentrations depends on subtle changes in insulin synthesis and secretion by beta cells and on their capacity for large increases in secretion after meals, requiring large stores of insulin (Tarabra et al., 2012). It is critical that islets maintain adequate beta cell mass in response to various fluctuations in demand (Tarabra et al., 2012). Beta cell mass is enhanced by proliferation (replication of beta cells), neogenesis (differentiation from non-beta cells), hyperplasia (increased beta cell number) and hypertrophy (increased beta cell size), and is decreased by beta cell death; through apoptosis, necrosis, autophagy, and ferroptosis; hypoplasia (decreased beta cell number); and hypotrophy (decreased beta cell size). The expansion and demise of beta cell mass through stimulants and insults respectively are likely triggered through one or more of these processes of beta cell replenishment (beta cell expansion) and death (beta cell demise).

Proliferation refers to an increase in beta cells from beta cell replication (beta cell self-replenishment) whereas beta cell hyperplasia occurs by beta cell replication or beta cell neogenesis from non-beta cells. Both beta cell replication and neogenesis contribute to the expansion of beta cell mass and require external stimuli such as hormones and growth factors (Bouwens and Rooman, 2005). Beta cells are dynamic and altered in response to fluctuating metabolic demand for insulin. Beta cell hypertrophy and hyperplasia occur during beta cell compensation to increase beta cell mass in response to hyperglycemia in diabetogenic states (Cerf et al., 2012). In several insulin resistant and diabetic rodent models, most islets were mildly enlarged and displayed hypertrophy and hyperplasia (Jones et al., 2010). Further, beta cell hypertrophy contributes to beta cell compensation in high fat diet-induced insulin resistance and the master beta cell transcription factor, pancreatic duodenal homeobox 1 (Pdx1), regulates beta cell size (Sachdeva et al., 2009), i.e., Pdx1 influences beta cell hyper- or hypotrophy. In contrast, beta cell hypotrophy results from beta cell death via various processes and insults and contributes to reduced beta cell mass. In addition, beta cell hypotrophy was found in hyperglycemic weanling rats exposed to a high fat diet during any single week of gestation (Cerf et al., 2007). Hyperglycemia may be exacerbated by the inability of hypotrophic and hypoplastic beta cells to synthesize and secrete sufficient insulin which consequently results in hypoinsulinemia (Cerf et al., 2007).

In diabetes, reduced beta cell mass occurs through apoptosis, necrosis, autophagy, and potentially ferroptosis. In human type 2 diabetes, both increased apoptosis and reduced replication may contribute to beta cell loss and reduced beta cell mass (Karaca et al., 2009). Beta cell hyperplasia and hyperinsulinemia compensate for progressively increasing insulin resistance to maintain normoglycemia; with time apoptosis exceeds the rate of replication and beta cell mass declines (Kiraly et al., 2008). The cytokine, IL1, induces beta cell necrosis suggesting that macrophage-derived cytokines participate in the initial pathogenesis of diabetes by inducing beta cell death by a mechanism that promotes necrosis and islet inflammation (Steer et al., 2006). Autophagy, a catabolic process that involves the degradation of cellular components through the lysosomal machinery, is important for maintaining normal islet homeostasis and compensatory beta cell hyperplasia in response to high fat dietary intake (Ebato et al., 2008). In type 2 diabetic patients, increased beta cell death was associated with altered autophagy suggesting that autophagy can be induced by metabolic perturbations (Marchetti and Masini, 2009). The cell death process of ferroptosis is morphologically, biochemically, and genetically distinct from apoptosis, various forms of necrosis and autophagy, and is characterized by iron-dependent accumulation of lethal lipid ROS (Dixon et al., 2012). The role of ferroptosis in beta cell demise still requires elucidation.

Beta cells initially compensate for the insulin resistance associated with obesity by increasing insulin secretion (Kasuga, 2006). When beta cell loss reaches the point of causing hyperglycemia, the beta cell replication rate is presumably maximally stimulated; therefore a further elevation in glucose concentrations will not increase replication (Porat et al., 2011). Glucose homeostasis maintains normoglycemia by adapting the mass and function of beta cells that counter insulin resistance, reduced beta cell mass, and excess nutrition (Liu et al., 2001).

### Glucose potentiates beta cell proliferation

The physiology of beta cells and insulin sensitive tissues are associated and mediated by metabolites, notably glucose. The beta cells compensate (adapt) in response to fluctuations in the demand for insulin. In states of increased insulin demand such as hyperglycemia, beta cells compensate to restore glucose homeostasis (maintain normoglycemia) via beta cell hypertrophy, and hyperplasia (to collectively increase beta cell mass) (Cerf, 2010) also increasing insulin biosynthesis (Brissova et al., 2005). Glucose increases the rate of beta cell proliferation *in vitro* (Kwon et al., 2004) and during short periods of glucose infusion in rodents (Bonner-Weir et al., 1989; Paris et al., 2003; Alonso et al., 2007) with glucose identified as the key systemic factor controlling beta cell replication (Porat et al., 2011). All beta cells appear to equally contribute to growth and maintenance (Brennand et al., 2007).

### Beta cell trajectories

Beta cell mass is dynamic and can respond to environmental cues such as glucose and insulin (Paris et al., 2003). Beta cell number increases markedly in the first year of rodent life (Montanya et al., 2000; Dor et al., 2004), up to 1.5-fold during pregnancy (Van Assche et al., 1978; Scaglia et al., 1995) and up to 10-fold in insulin resistant states (Kulkarni et al., 2004).

When not constrained by persistent autoimmune attack or the toxicity of persistent hyperglycemia (glucotoxicity) (Ryu et al., 2001), beta cells inherently have the capacity to regenerate (Brennand et al., 2007). While the mechanism regulating beta cell expansion remains unclear, all beta cells are capable of replication (Brennand et al., 2007). Pluripotent stem cells also serve as sources of new beta cells. This however requires their defined terminal differentiation into functional beta cells which presents a major challenge.

In rodents, beta cell replication rates in young adult mice (12 week old) are highly variable from 2% (Finegood et al., 1995) to 15% per day (Teta et al., 2005). It was assumed that if 5% of beta cells replicate per day, and that all beta cells are equivalent, beta cells should divide approximately every 20 days (Brennand et al., 2007). However, using the range of 2–15% beta cell replication per day, and factoring in the median of a 8.5% replication rate, it is possible that beta cells can divide every 12 days, in physiological states, i.e., in the absence of any persistent hyperglycemic excursions. Altered metabolic states requiring increased insulin demand will alter beta cell replication rates.

Beta cell replication varies in humans and rodents. Human beta cells, unlike young rodent beta cells, are long-lived with little adaptive change during adulthood and are largely established by 20 years (Cnop et al., 2010). In humans, an age-related decrease of replicating cells reflects decreased adaptability (Reers et al., 2009). In young rodents (<1 year), the half-life of beta cells is estimated at 30–60 days (Finegood et al., 1995). In human adult beta cells, replication is estimated at 10-fold less than in adult mice (Butler et al., 2003, 2007) with the most replication in <5 year old children (Meier et al., 2008).

### Beta cell proliferation: Mechanisms

During neonatal life, the endocrine pancreas undergoes substantial remodeling, which involves significant apoptosis, replication, and neogenesis of islet cells (Scaglia et al., 1997; Petrik et al., 1998; Bonner-Weir et al., 2010). Islet mass grows into adulthood to match increased hormonal demand (Zhang et al., 2012). In contrast, there is little change in islet mass in adults except in response to physiological and/or pathological changes such as pregnancy and obesity (Parsons et al., 1992; Weir et al., 2001), while adaptive islet cell proliferation is severely restricted in aged mice (Rankin and Kushner, 2009).

By GSIS, beta cells tightly regulate systemic glycemia within a narrow range. Insufficient functional beta cell mass is the causal factor of type 1 and a major contributor to type 2 diabetes, emphasizing the importance of understanding beta cell dynamics (Butler et al., 2003; Muoio and Newgard, 2008). In healthy adult mice, and in mice recovering from a diabetogenic injury, new beta cells are derived mainly by replication of pre-existing beta cells (Dor et al., 2004; Georgia and Bhushan, 2004; Brennand et al., 2007; Nir et al., 2007; Teta et al., 2007; Meier et al., 2008).

Signals controlling beta cell number may therefore act by modulating the survival and/or proliferation of differentiated beta cells (Porat et al., 2011). Food intake or glucose infusion increases beta cell replication in mice (Chick and Like, 1971; Chick, 1973; Bonner-Weir et al., 1989; Alonso et al., 2007) and insulin resistance results in a compensatory increase in beta cell mass (Kulkarni et al., 2004). There is evidence that systemic factors control beta cell proliferation, including the observation of beta cell hyperplasia in mice lacking insulin receptors in the liver (Michael et al., 2000; Kulkarni et al., 2004; Okada et al., 2007; Imai et al., 2008), and the demonstration that systemic factors in insulin resistant animals induce beta cell proliferation in islet grafts (Flier et al., 2001).

Uncontrolled beta cell proliferation will increase beta cell numbers and enhance the functional capacity. However, hypoglycemia could be a consequence of increased, uncontrolled beta cell proliferation due to an excess of beta cells, and the resultant over secretion of insulin. Further, increased beta cell numbers will occur at the expense of other islet cell types that secrete hormones antagonistic to insulin to also facilitate glucose homeostasis. Disrupting the islet cell population will limit the ability for maintaining glucose homeostasis. Apoptosis may also be potentiated to regulate uncontrolled proliferation. The balance between sustaining proliferation and evading apoptosis is critical for the initial beta cell compensatory response to normalize glycemia. This delicate shift to either physiological process has to adapt to metabolic demand. If this balance is disrupted to favor either process it would exacerbate the diabetogenic state.

Glucose can increase the rate of beta cell proliferation *in vitro* (Kwon et al., 2004) and during short periods of glucose infusion *in vivo* (rodents) (Bonner-Weir et al., 1989; Paris et al., 2003; Alonso et al., 2007). Insulin, FFA, and incretin hormones have also been proposed as beta cell proliferative agents, particularly in insulin resistant states where circulating glucose concentrations are not measurably elevated but several other beta cell mitogens are yet to be identified (Porat et al., 2011).

A recent study identified a simple mechanism for homeostasis of beta cell proliferation and mass where beta cells adjust their proliferation rate according to the rate of glycolysis; this provides a system for sensitive measurement of organismal demand for beta cells, while normoglycemia is maintained (Porat et al., 2011). The same homeostatic mechanism appears to be responsible for the control of beta cell number during healthy adult life and during regeneration following injury (Porat et al., 2011). Physiologically, this model explains how beta cell number can be finely adjusted according to the organism’s needs without deviating from the normoglycemic range (Porat et al., 2011).

The capacity of beta cells to proliferate in response to insulin resistance is critical for glucose homeostasis and for preventing the progression of type 2 diabetes (Blandino-Rosano et al., 2012). Proliferation of mature beta cells is one of the components responsible for maintenance of beta cell mass in adult life (Dor et al., 2004). A highly perfused subpopulation of islets demonstrated a higher rate of beta cell proliferation (Lau et al., 2012). Islet endothelial paracrine factors can increase beta cell proliferation (Lau et al., 2012) and the transcription factor, Islet 1, has been identified as crucial for sustained beta cell proliferation and the prevention of apoptosis in postnatal beta cells (Du et al., 2009). Tcf7l2 promotes beta cell proliferation, protects against apoptosis, and improves insulin secretion (Loder et al., 2008; Shu et al., 2008, 2009).

Beta cell proliferation progressively reduces with age. Adaptive beta cell proliferation is severely restricted with advanced age (Rankin and Kushner, 2009). In mice, beta cell regeneration, which occurs mainly by self-renewal, is severely and abruptly restricted by middle age (Rankin and Kushner, 2009). The regenerative capacity of adult beta cells becomes limited by early middle age (1 year, about 40% of the life span in mice) (Harrison and Archer, 1987). Beta cell replication may become fully restricted when adult insulin requirements are established in middle age (Rankin and Kushner, 2009). In human islets from donors aged 17–74 years, there was an age-related decreased expression of Pdx1 indicating both a decreased capacity with age for cellular proliferation (decreased plasticity) and reduced insulin formation and secretion (Maedler et al., 2006). A significantly higher incidence of beta cell neogenesis was observed in the premature and developing human pancreas (Gregg et al., 2012). Postnatally, human beta cell neogenesis was observed at a negligible rate of ≤0.5% in normal human pancreata with several rare instances of beta cell neogenesis observed at 1, 15, and 39 years (Gregg et al., 2012).

## Beta Cell Preservation

### Determinants of beta cell preservation

Several recently identified factors regulate beta cells. These factors are involved in beta cell maintenance (mass, compensation, and function) and ultimately contribute to beta cell preservation. Beta cell preservation refers to the maintenance of both beta cell structure (integrity or architecture including beta cell mass, number, and size) and function (beta cell physiology).

Serum response factor (SRF), an essential regulator of myogenic and neurogenic genes, is highly enriched in beta cells and the insulin gene is a target of SRF (Sarkar et al., 2011). Insulin gene expression is glucose responsive; SRF is a glucose concentration sensitive regulator of insulin gene expression and may therefore play an important role in glucose homeostasis (Sarkar et al., 2011).

Alpha motif domain containing 4b/harmonin-interacting, ankyrin repeat-containing protein (Anks4b/Harp) is a target of HNF4α in beta cells (Sato et al., 2012). Anks4b interacts with glucose regulated protein 78 (GRP78), a major chaperone protein that protects cells from ER stress *in vitro* and *in vivo* (Sato et al., 2012). HNF4α plays an important role in the regulation of ER stress and apoptosis in beta cells (Sato et al., 2012). HNF4α and HNF1α regulate common target genes through interdependent regulatory mechanisms (Boj et al., 2010). Although the mechanism of the functional interaction between HNF4α and HNF1α is still unclear, Anks4b gene expression represents another example of such interdependent regulation (Sato et al., 2012). Anks4b is a target of HNF4α and uncovers a new role for this transcription factor in regulating beta cell susceptibility to ER stress (Sato et al., 2012).

Recently it was shown that uncoupling protein 2 (UCP2) does not contribute to mitochondrial uncoupling in beta cells in intact islets (Robson-Doucette et al., 2011). However, there is growing evidence that implicates mitochondrial dysfunction in the pathophysiology of beta cell secretory failure (Mulder and Ling, 2009). Beta cell mitochondria serve as the fuel integrators of beta cells and generate signals for insulin secretion, the principal being ATP (Wikstrom et al., 2012). Therefore mitochondrial function predicts beta cell function as ATP is a key trigger for GSIS. The respiratory profile of human islets prior to transplantation into animal models may predict the transplantation outcome (Sweet et al., 2005, 2008; Papas et al., 2007); i.e., islets with high levels of oxygen consumption were more likely to reverse diabetes (Wikstrom et al., 2012).

The survival of beta cells in type 2 diabetes is particularly important as the disease is characterized by a progressive loss of beta cell numbers due to glucolipotoxicity (Shimabukuro et al., 1998; El-Assaad et al., 2003) and other insults. MIN6 cells express constitutively active peroxisome proliferator-activated receptor (PPARγ) in the presence of high glucose (Rao et al., 2012). More interestingly however, the reversal of the effect by glucose and adiponectin to induce insulin release, provides a novel pathway by which adiponectin accentuates insulin secretion in MIN6 cells and increases insulin content (Rao et al., 2012).

Hepatocyte growth factor (HGF), the growth inhibitory peptide or glucose-dependent insulinotropic polypeptide (GIP), the transcription factor paired box gene (Pax4), and the orphan nuclear liver receptor homolog 1 (LRH1) are factors that exert several beneficial actions on beta cells simultaneously such as conferring beta cell protection and enhancing proliferation (Mellado-Gil et al., 2012). In addition, some of these factors have been shown to improve beta cell function under pathophysiological conditions (Mellado-Gil et al., 2012). HGF and Pax4 both inhibit the NF-κB pathway leading to enhanced beta cell survival (Mellado-Gil et al., 2012). Similarly, GIP and Pax4 enhance expression of the anti-apoptotic gene *bcl2* (Mellado-Gil et al., 2012). HGF and Pax4 also promote trans-differentiation of pancreatic ductal endothelial cells and alpha cells into beta cells (Mellado-Gil et al., 2012). This could be extremely beneficial as an alternative method to replenish functional beta cell mass due to the limited replication capacity of beta cells (Mellado-Gil et al., 2012). It is also interesting that alpha cells appear less susceptible to autoimmune attack with an apparent increase in the number of alpha cells in type 2 diabetic patients (Mellado-Gil et al., 2012). In this context, a combined HGF/c-met, Pax4, and GIP therapy could be optimal: beta cell protection with increased proliferation with the generation of new beta cells from alpha cells (Mellado-Gil et al., 2012).

The preservation of beta cell mass is crucial for islets to maintain normal glucose concentrations in the face of increasing glycemia and decreasing insulin sensitivity (Rao et al., 2012). The functional significance of activating PPARγ using thiazolidinedione (TZD) therapy in type 2 diabetes is to prevent beta cell apoptosis as these compounds enhance beta cell survival in diabetic patients (Ovalle and Bell, 2002; Pelzer et al., 2005). The PPARγ agonist rosiglitazone prevents beta cell death in *db/db* mice and Zucker diabetic fatty (ZDF) rats (Finegood et al., 2001). However, the critical determinant is whether these preserved beta cells are fully functional, have compromised function or are redundant. Preserving beta cells that are not fully functional, i.e., respond properly and promptly to insulin demands, will compound beta cell dysfunction and insulin resistance. These cells will occupy the space of fully functional beta cells thereby compromising collective insulin synthesis and secretion.

Because replenishing lost beta cells provides a potential cure for diabetes, identifying molecules capable of inducing endocrine cell differentiation in animal models and elucidating their mechanism of action are particularly valuable for diabetes research (Wang et al., 2008). The transcription factors, myelin transcription factor (Myt1) and neurogenin (Ngn3), form a feed-forward expression loop to promote the differentiation of most if not all endocrine cells (Wang et al., 2008) and therefore present a starting point for beta cell replenishment.

Some physiological processes also prime beta cells for survival such as dedifferentiation and autophagy. To avoid demise, beta cells dedifferentiate, and at a later stage, resume their beta cell identity and function. Beta cell dedifferentiation is the regression to an endocrine progenitor-like stage rather than a degenerative stage (Talchai et al., 2012). By adopting the dedifferentiated fate, beta cells enhance their survival (Talchai et al., 2012). There is ample time for transition between functional depletion of insulin in beta cells and their demise that may allow the salvaging of dedifferentiated beta cells; this presents a novel approach for treating beta cell dysfunction in diabetes (Talchai et al., 2012).

Autophagy is a pathway for cells to degrade organelles and is an adapted mechanism for cells to reduce ROS (Scherz-Shouval and Elazar, 2011) and eliminate ER stress (Ding et al., 2007). This catabolic process involves the degradation of cellular components through the lysosomal machinery and is important for maintaining normal islet homeostasis and compensatory beta cell hyperplasia (Ebato et al., 2008). Autophagy plays a key role in the protection of beta cells from apoptosis, stabilizing beta cell structure, and maintaining beta cell function (Hur et al., 2010; Las and Shirihai, 2010). However, in human type 2 diabetes, autophagy can also contribute to loss of beta cell mass (Masini et al., 2009). Thus, whether autophagy plays a protective role or harms beta cells requires further investigation (Wu et al., 2013).

### Novel treatments

Treatments for beta cell dysfunction should strive to ameliorate beta cell structure and/or function, stimulate beta cell proliferation to enhance beta cell mass and thereby allow compensation to restore and/or maintain beta cell function. This ultimately maintains glucose homeostasis. A recent study emphasized the potential importance of analyzing molecular pathways to identify potential therapeutic targets (Koulmanda et al., 2012). Therapeutic strategies aimed at repopulating insulin-producing cells show great potential for restoring glycemia in diabetes (Feng et al., 2012). Factors should not be studied in isolation but rather collectively to mimic physiology.

c-Kit is important in the development and function of islets, especially in support of beta cell proliferation, maturation, and survival (Oberg et al., 1994; Oberg-Welsh and Welsh, 1996; LeBras et al., 1998; Welsh et al., 2000; Rachdi et al., 2001; Yashpal et al., 2004; Li et al., 2007). c-Kit overexpression in beta cells confers improved glucose metabolism by enhancing insulin secretion and increases beta cell mass and proliferation, probably through the activation of the phosphatidylinositol-3-kinase (PI3K)-Akt signaling pathway (Feng et al., 2012).

Mimitin, a small 20 kDa mitochondrial protein and direct target of c-myc, is involved in cell proliferation (Tsuneoka et al., 2005) and can act as a modulator of GSIS and prevent its inhibition by proinflammatory cytokines (Hanzelka et al., 2012).

Adiponectin is capable of increasing MIN6 cell proliferation (Rao et al., 2012). Adiponectin increased MIN6 cell number in 25 mM glucose but had no effect on MIN6 cell proliferation in 3 mM glucose (Rao et al., 2012). This demonstrated that hyperglycemia was required for increasing proliferation. Adiponectin also has positive effects on MIN6 cell proliferation via pathways unrelated to PPARγ activation (Rao et al., 2012).

Exenatide, a glucagon-like peptide-1 receptor (GLP-1R) agonist, enhances beta cell function and survival during culture conditions used in clinical islet transplantation which presents a feasible approach to preserve beta cells during pre-transplant islet culture (Park et al., 2013).

Epoxyoukalide, a marine product, protected against basal and cytokine-induced apoptosis, maintained beta cell function, and induced beta cell proliferation mediated by extracellular signal-regulated kinases (ERK1/2) activation and targets, cyclin D2, and cyclin R (Lopez-Acosta et al., 2013).

Insulin secretion in the presence of resveratrol is increased in various pancreatic beta cell cultures (Chen et al., 2007). Phenolic compounds potentially positively preserve beta cells and their function (Lin et al., 2012). Quercetin and naringenin possibly protect beta cells from cytokine-induced toxicity by enhancing cell survival through the PI3K pathway, independent of p-p38 mitogen-activated protein kinases (MAPK), or inducible NO synthase (iNOS) as demonstrated in INS-1 cells (Lin et al., 2012). In addition, quercetin and naringenin induce pAkt activation and could further participate in beta cell protection from cytokine-induced cell apoptosis, possibly by a NO-independent mechanism and could be potent agents to benefit beta cell mass preservation (Lin et al., 2012).

### Healthy diet and regular exercise

A healthy normocaloric diet that is well balanced, limited in saturated fat content, and meets the recommended daily allowances of key nutrients protects beta cells and enhances longevity in healthy individuals. Reduced energy intake combined with exercise improves insulin sensitivity (Weickert, 2012). Additional dietary measures that reduce insulin resistance include ingesting a Mediterranean dietary pattern that avoids excess dietary fat intake; substituting saturated fatty acids and trans fatty acids with monounsaturated fatty acids and polyunsaturated fatty acids, emphasizing cereal fiber content in the diet and when on a high protein diet, maintaining high levels of exercise (Weickert, 2012). Patients with poorly controlled type 2 diabetes who were placed on a hypocaloric diet for up to 12 weeks experienced a marked and rapid decrease in liver fat content (85%), associated specifically with normalization in hepatic insulin sensitivity and reductions in fasting hyperglycemia and hepatic glucose production without changes in intramyocellular lipid or insulin-mediated whole body glucose disposal (Petersen et al., 2005).

Regular exercise, although it may not necessarily reduce weight, enhances insulin sensitivity. Importantly, exercise concomitant with weight loss improves beta cell function (Dela et al., 2004; Solomon et al., 2010). Exercise maintained or enhanced beta cell function in older obese individuals, with improved beta cell function correlating with reduced glucose concentrations (Malin and Kirwan, 2012). In the ZDF rat, voluntary running prevented the development of diabetes despite continuing hyperphagia, obesity, and hyperlipidemia (Delghingaro-Augusto et al., 2012). Compensatory insulin secretion was preserved and hyperglycemia prevented by exercise was characterized by enhanced insulin secretion per islet and the prevention of severe depletion of islet insulin stores (Delghingaro-Augusto et al., 2012). Frequent exercise and adopting a healthy diet is a strategy to improve diabetic outcomes, particularly in pre-diabetic states such as obesity and/or insulin resistance and beta cell dysfunction.

## Beta Cell Proliferation and Compensation Counters Beta Cell Demise and Dysfunction but Promotes Beta Cell Preservation

**Figure 2:**
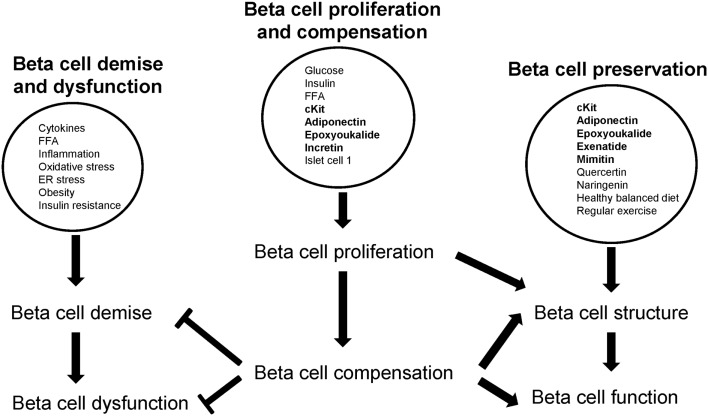
**Beta cell proliferation and compensation counters beta cell demise and dysfunction but promotes beta cell survival**. Factors are encircled; proliferating factors are in bold.

Cytokine-mediated oxidative stress and inflammation, inherent in obesity and insulin resistance, induces beta cell death therefore the beta cell population declines contributing to the manifestation of beta cell dysfunction (Figure 2). Similarly peripheral FFA-induced inflammation, as in obesity and insulin resistance, leads to beta cell destruction and dysfunction (Figure 2). Further, within the pancreas, saturated FFA induce islet inflammation and increase cytokine expression in beta cells thereby inducing beta cell dysfunction.

Metabolites, hormones, and transcription factors stimulate beta cell proliferation to sustain beta cell compensation (Figure 2). Beta cell compensation is a critical intervention process: it both inhibits beta cell demise and dysfunction; and protects beta cell structure and function (Figure 2).

Proliferative agents, novel compounds, and healthy lifestyles help to preserve beta cells by maintaining beta cell integrity and function (Figure 2). Therefore with an adequate (and functional) beta cell population, beta cell physiology is maintained. Apart from feeding beta cell compensation, beta cell proliferation also restores beta cell population (when replenishment > death) thereby contributing to beta cell maintenance.

## Perspectives on Beta Cell Dysfunction and Insulin Resistance

Beta cell dysfunction signals an advanced state of diabetes as insufficient insulin is secreted to meet demand. Insulin resistance precedes the pathogenesis for several modern diseases (Samuel and Shulman, 2012). Thus understanding the pathogenesis of insulin resistance has become increasingly important to guide the development of future therapies and inform health and economic policy (Samuel and Shulman, 2012).

**Figure 3:**
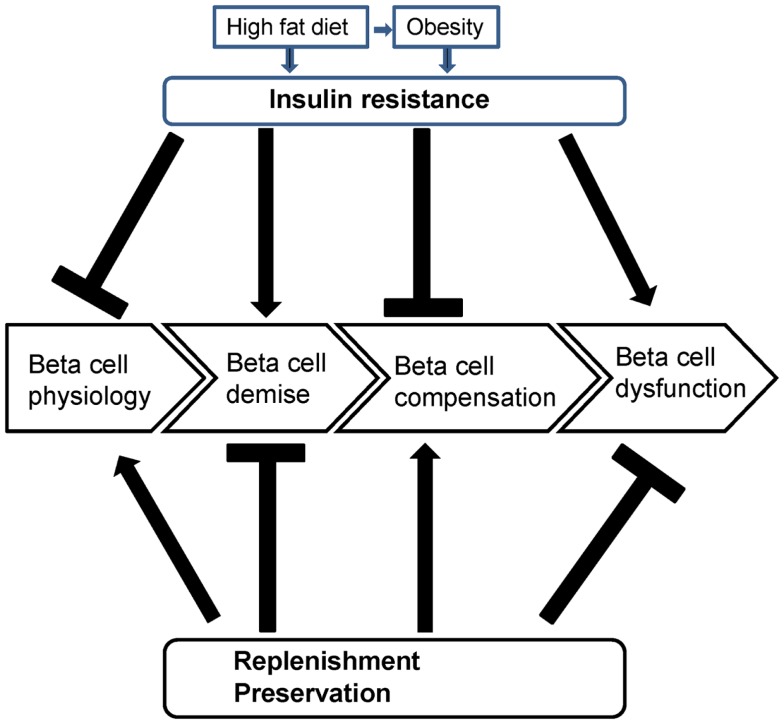
**Beta cell dysfunction and insulin resistance dynamics**.

The relationship between insulin resistance and beta cell dysfunction is dynamic and largely dependent on the metabolic state that is primarily determined by glycemic status and consequently insulinemic status. Both a high fat diet and obesity trigger insulin resistance independently, with a high fat diet contributing to overweight and obesity (Figure 3). In the etiology of beta cell dysfunction, firstly, beta cell physiology is maintained in healthy individuals (Figure 3). However, glucolipotoxicity and proinflammatory cytokines induce oxidative stress leading to beta cell demise, although other stressors exist (Figure 3). Beta cell compensation occurs when beta cell integrity is diminished. If beta cell compensation is successful, beta cell physiology is maintained (Figure 3). However if beta cell compensation is exhausted, beta cell dysfunction ensues (Figure 3). Insulin resistance impairs beta cell physiology and compensation thereby inducing beta cell demise and dysfunction. Beta cell replenishment and preservation, through novel agents, healthy lifestyles of balanced diets and regular exercise, maintains beta cell physiology, and compensation (when necessary) therefore protecting against beta cell demise and dysfunction (Figure 3). The main intervention strategy is to maintain sufficient beta cell compensation to restore and maintain beta cell physiology to avoid beta cell dysfunction and the subsequent progression to diabetes. This is achieved by maintaining adequate beta cell preservation, i.e., by preserving beta cell structure and function, and replenishment by lifestyle and, if necessary, therapeutic interventions.

Beta cell physiology should be preserved throughout life but is adversely impacted with aging and altered metabolic states such as obesity that requires a sustained increase in insulin. Insulin resistance promotes beta cell demise and inhibits beta cell compensation which thereby promotes beta cell dysfunction. Replenishing or preserving beta cells maintains beta cell physiology and allows for beta cell compensation which combats beta cell demise and beta cell dysfunction. The preservation of beta cells by replenishment to mitigate against insults and maintain beta cell physiology will improve the metabolic outcomes associated with diabetes.

## Conflict of Interest Statement

The authors declare that the research was conducted in the absence of any commercial or financial relationships that could be construed as a potential conflict of interest.

## References

[B1] Alonso L. C. Yokoe T. Zhang P. Scott D. K. Kim S. K. O’Donnell C. P. (2007). Glucose infusion in mice: a new model to induce beta-cell replication. Diabetes 56, 1792–1801 10.2337/db06-1513 17400928 PMC2921922

[B2] Ashcroft F. M. Rorsman P. (2012). Diabetes mellitus and the beta cell: the last ten years. Cell 148, 1160–1171 10.1016/j.cell.2012.02.010 22424227 PMC5890906

[B3] Bergman R. N. (2005). Minimal model: perspective from 2005. Horm. Res. 64(Suppl. 3), 8–15 10.1159/000089312 16439839

[B4] Blandino-Rosano M. Alejandro E. U. Sathyamurthy A. Scheys J. O. Gregg B. Chen A. Y. (2012). Enhanced beta cell proliferation in mice overexpressing a constitutively active form of Akt and one allele of p21 (Cip). Diabetologia 55, 1380–1389 10.1007/s00125-012-2465-9 22327314 PMC3646796

[B5] Boj S. F. Petrov D. Ferrer J. (2010). Epistasis of transcriptomes reveals synergism between transcriptional activators Hnf1alpha and Hnf4alpha. PLoS Genet. 6:e1000970 10.1371/journal.pgen.1000970 20523905 PMC2877749

[B6] Bonnefond A. Froguel P. Vaxillaire M. (2010). The emerging genetics of type 2 diabetes. Trends Mol. Med. 16, 407–416 10.1016/j.molmed.2010.06.004 20728409

[B7] Bonner-Weir S. Deery D. Leahy J. L. Weir G. C. (1989). Compensatory growth of pancreatic beta-cells in adult rats after short-term glucose infusion. Diabetes 38, 49–53 10.2337/diabetes.38.1.49 2642434

[B8] Bonner-Weir S. Li W. C. Ouziel-Yahalom L. Guo L. Weir G. C. Sharma A. (2010). Beta-cell growth and regeneration: replication is only part of the story. Diabetes 59, 2340–2348 10.2337/db10-0084 20876724 PMC3279552

[B9] Bouwens L. Rooman I. (2005). Regulation of pancreatic beta-cell mass. Physiol. Rev. 85, 1255–1270 10.1152/physrev.00025.2004 16183912

[B10] Brennand K. Huangfu D. Melton D. (2007). All beta cells contribute equally to islet growth and maintenance. PLoS Biol. 5:e163 10.1371/journal.pbio.0050163 17535113 PMC1877817

[B11] Brissova M. Blaha M. Spear C. Nicholson W. Radhika A. Shiota M. (2005). Reduced PDX-1 expression impairs islet response to insulin resistance and worsens glucose homeostasis. Am. J. Physiol. Endocrinol. Metab. 288, 707–714 10.1152/ajpendo.00252.2004 15562255

[B12] Butler A. E. Janson J. Bonner-Weir S. Ritzel R. Rizza R. A. Butler P. C. (2003). Beta-cell deficit and increased beta-cell apoptosis in humans with type 2 diabetes. Diabetes 52, 102–110 10.2337/diabetes.52.9.2304 12502499

[B13] Butler P. C. Meier J. J. Butler A. E. Bhushan A. (2007). The replication of beta cells in normal physiology, in disease and for therapy. Nat. Clin. Pract. Endocrinol. Metab. 3, 758–768 10.1038/ncpendmet0647 17955017

[B14] Cerf M. E. (2010). High fat programming of beta-cell failure. Adv. Exp. Med. Biol. 654, 77–89 10.1007/978-90-481-3271-3_5 20217495

[B15] Cerf M. E. Chapman C. S. Louw J. (2012). High-fat programming of hyperglycemia, hyperinsulinemia, insulin resistance, hyperleptinemia, and altered islet architecture in 3-month-old wistar rats. ISRN Endocrinol. 2012, 627270 22988521 10.5402/2012/627270 PMC3440883

[B16] Cerf M. E. Williams K. Chapman C. S. Louw J. (2007). Compromised beta-cell development and beta-cell dysfunction in weanling offspring from dams maintained on a high-fat diet during gestation. Pancreas 34, 347–353 10.1097/MPA.0b013e31802ee9ae 17414058

[B17] Chen W. P. Chi T. C. Chuang L. M. Su M. J. (2007). Resveratrol enhances insulin secretion by blocking K(ATP) and K(V) channels of beta cells. Eur. J. Pharmacol. 568, 269–277 10.1016/j.ejphar.2007.04.047 17573071

[B18] Chick W. L. (1973). Beta cell replication in rat pancreatic monolayer cultures. Effects of glucose, tolbutamide, glucocorticoid, growth hormone and glucagon. Diabetes 22, 687–693 4125576 10.2337/diab.22.9.687

[B19] Chick W. L. Like A. A. (1971). Effects of diet on pancreatic beta cell replication in mice with hereditary diabetes. Am. J. Physiol. 221, 202–208 4933588 10.1152/ajplegacy.1971.221.1.202

[B20] Choi H. J. Hwang S. Lee S. H. Lee Y. R. Shin J. Park K. S. (2012). Genome-wide identification of palmitate-regulated immediate early genes and target genes in pancreatic beta-cells reveals a central role of NF-kappaB. Mol. Biol. Rep. 39, 6781–6789 10.1007/s11033-012-1746-1 22302392

[B21] Cnop M. Hughes S. J. Igoillo-Esteve M. Hoppa M. B. Sayyed F. van de Laar L. (2010). The long lifespan and low turnover of human islet beta cells estimated by mathematical modelling of lipofuscin accumulation. Diabetologia 53, 321–330 10.1007/s00125-009-1562-x 19855953

[B22] Cnop M. Welsh N. Jonas J. C. Jorns A. Lenzen S. Eizirik D. L. (2005). Mechanisms of pancreatic beta-cell death in type 1 and type 2 diabetes: many differences, few similarities. Diabetes 54(Suppl. 2), S97–S107 10.2337/diabetes.54.suppl_2.S97 16306347

[B23] Coelho D. F. Pereira-Lancha L. O. Chaves D. S. Diwan D. Ferraz R. Campos-Ferraz P. L. (2011). Effect of high-fat diets on body composition, lipid metabolism and insulin sensitivity, and the role of exercise on these parameters. Braz. J. Med. Biol. Res. 44, 966–972 10.1590/S0100-879X2010007500120 21876873

[B24] Collins S. C. Hoppa M. B. Walker J. N. Amisten S. Abdulkhader F. Bengtsson M. (2010). Progression of diet-induced diabetes in C57Bl6J mice involves functional dissociation of Ca2^+^ channels from secretory vesicles. Diabetes 59, 1192–1201 10.2337/db09-0791 20150285 PMC2857899

[B25] Del Guerra S. Lupi R. Marselli L. Masini M. Bugliani M. Sbrana S. (2005). Functional and molecular defects of pancreatic islets in human type 2 diabetes. Diabetes 54, 727–735 10.2337/diabetes.54.3.727 15734849

[B26] Dela F. von Linstow M. E. Mikines K. J. Galbo H. (2004). Physical training may enhance beta-cell function in type 2 diabetes. Am. J. Physiol. Endocrinol. Metab. 287, E1024–E1031 10.1152/ajpendo.00056.2004 15251867

[B27] Delghingaro-Augusto V. Decary S. Peyot M. L. Latour M. G. Lamontagne J. Paradis-Isler N. (2012). Voluntary running exercise prevents beta-cell failure in susceptible islets of the Zucker diabetic fatty rat. Am. J. Physiol. Endocrinol. Metab. 302, E254–E264 10.1152/ajpendo.00360.2011 22045312

[B28] Ding W. X. Ni H. M. Gao W. Yoshimori T. Stolz D. B. Ron D. (2007). Linking of autophagy to ubiquitin-proteasome system is important for the regulation of endoplasmic reticulum stress and cell viability. Am. J. Pathol. 171, 513–524 10.2353/ajpath.2007.070188 17620365 PMC1934546

[B29] Dixon S. J. Lemberg K. M. Lamprecht M. R. Skouta R. Zaitsev E. M. Gleason C. E. (2012). Ferroptosis: an iron-dependent form of nonapoptotic cell death. Cell 149, 1060–1072 10.1016/j.cell.2012.03.042 22632970 PMC3367386

[B30] Donath M. Y. Boni-Schnetzler M. Ellingsgaard H. Halban P. A. Ehses J. A. (2010). Cytokine production by islets in health and diabetes: cellular origin, regulation and function. Trends Endocrinol. Metab. 21, 261–267 10.1016/j.tem.2009.12.010 20096598

[B31] Donath M. Y. Shoelson S. E. (2011). Type 2 diabetes as an inflammatory disease. Nat. Rev. Immunol. 11, 98–107 10.1038/nri2925 21233852

[B32] Dor Y. Brown J. Martinez O. I. Melton D. A. (2004). Adult pancreatic beta-cells are formed by self-duplication rather than stem-cell differentiation. Nature 429, 41–46 10.1038/nature02520 15129273

[B33] Drews G. Krippeit-Drews P. Dufer M. (2010). Oxidative stress and beta-cell dysfunction. Pflugers Arch. 460, 703–718 10.1007/s00424-010-0862-9 20652307

[B34] Du A. Hunter C. S. Murray J. Noble D. Cai C. L. Evans S. M. (2009). Islet-1 is required for the maturation, proliferation, and survival of the endocrine pancreas. Diabetes 58, 2059–2069 10.2337/db08-0987 19502415 PMC2731519

[B35] Dupuis J. Langenberg C. Prokopenko I. Saxena R. Soranzo N. Jackson A. U. (2010). New genetic loci implicated in fasting glucose homeostasis and their impact on type 2 diabetes risk. Nat. Genet. 42, 105–116 10.1038/ng0510-464a 20081858 PMC3018764

[B36] Ebato C. Uchida T. Arakawa M. Komatsu M. Ueno T. Komiya K. (2008). Autophagy is important in islet homeostasis and compensatory increase of beta cell mass in response to high-fat diet. Cell Metab. 8, 325–332 10.1016/j.cmet.2008.08.009 18840363

[B37] Eguchi K. Manabe I. Oishi-Tanaka Y. Ohsugi M. Kono N. Ogata F. (2012). Saturated fatty acid and TLR signaling link beta cell dysfunction and islet inflammation. Cell Metab. 15, 518–533 10.1016/j.cmet.2012.01.023 22465073

[B38] Eizirik D. L. Cnop M. (2010). ER stress in pancreatic beta cells: the thin red line between adaptation and failure. Sci. Signal. 3, e7 10.1126/scisignal.3110pe7 20179270

[B39] El-Assaad W. Buteau J. Peyot M. L. Nolan C. Roduit R. Hardy S. (2003). Saturated fatty acids synergize with elevated glucose to cause pancreatic beta-cell death. Endocrinology 144, 4154–4163 10.1210/en.2003-0410 12933690

[B40] Evans J. L. Goldfine I. D. Maddux B. A. Grodsky G. M. (2003). Are oxidative stress-activated signaling pathways mediators of insulin resistance and beta-cell dysfunction? Diabetes 52, 1–8 10.2337/diabetes.52.1.1 12502486

[B41] Feng Z. C. Li J. Turco B. A. Riopel M. Yee S. P. Wang R. (2012). Critical role of c-Kit in beta cell function: increased insulin secretion and protection against diabetes in a mouse model. Diabetologia 55, 2214–2225 10.1007/s00125-012-2566-5 22581040

[B42] Ferrannini E. (2010). The stunned beta cell: a brief history. Cell Metab. 11, 349–352 10.1016/j.cmet.2010.04.009 20444416

[B43] Finegood D. T. McArthur M. D. Kojwang D. Thomas M. J. Topp B. G. Leonard T. (2001). Beta-cell mass dynamics in Zucker diabetic fatty rats. Rosiglitazone prevents the rise in net cell death. Diabetes 50, 1021–1029 10.2337/diabetes.50.5.1021 11334404

[B44] Finegood D. T. Scaglia L. Bonner-Weir S. (1995). Dynamics of beta-cell mass in the growing rat pancreas. Estimation with a simple mathematical model. Diabetes 44, 249–256 10.2337/diabetes.44.3.249 7883109

[B45] Flier S. N. Kulkarni R. N. Kahn C. R. (2001). Evidence for a circulating islet cell growth factor in insulin-resistant states. Proc. Natl. Acad. Sci. U.S.A. 98, 7475–7480 10.1073/pnas.131192998 11404474 PMC34693

[B46] Florez J. C. (2008). Newly identified loci highlight beta cell dysfunction as a key cause of type 2 diabetes: where are the insulin resistance genes? Diabetologia 51, 1100–1110 10.1007/s00125-007-0891-x 18504548

[B47] Georgia S. Bhushan A. (2004). Beta cell replication is the primary mechanism for maintaining postnatal beta cell mass. J. Clin. Invest. 114, 963–968 10.1172/JCI22098 15467835 PMC518666

[B48] Gilbert E. R. Liu D. (2012). Epigenetics: the missing link to understanding beta-cell dysfunction in the pathogenesis of type 2 diabetes. Epigenetics 7, 841–852 10.4161/epi.21238 22810088 PMC3427279

[B49] Gregg B. E. Moore P. C. Demozay D. Hall B. A. Li M. Husain A. (2012). Formation of a human beta-cell population within pancreatic islets is set early in life. J. Clin. Endocrinol. Metab. 97, 3197–3206 10.1210/jc.2012-1206 22745242 PMC3431572

[B50] Gurgul-Convey E. Mehmeti I. Lortz S. Lenzen S. (2011). Cytokine toxicity in insulin-producing cells is mediated by nitro-oxidative stress-induced hydroxyl radical formation in mitochondria. J. Mol. Med. (Berl.) 89, 785–798 10.1007/s00109-011-0747-1 21487676

[B51] Hanzelka K. Skalniak L. Jura J. Lenzen S. Gurgul-Convey E. (2012). Effects of the novel mitochondrial protein mimitin in insulin-secreting cells. Biochem. J. 445, 349–359 10.1042/BJ20111920 22587331

[B52] Harrison D. E. Archer J. R. (1987). Genetic differences in effects of food restriction on aging in mice. J. Nutr. 117, 376–382 3559752 10.1093/jn/117.2.376

[B53] Henquin J. C. Dufrane D. Nenquin M. (2006). Nutrient control of insulin secretion in isolated normal human islets. Diabetes 55, 3470–3477 10.2337/db06-0868 17130494

[B54] Hoppa M. B. Collins S. Ramracheya R. Hodson L. Amisten S. Zhang Q. (2009). Chronic palmitate exposure inhibits insulin secretion by dissociation of Ca(2^+^) channels from secretory granules. Cell Metab. 10, 455–465 10.1016/j.cmet.2009.09.011 19945403 PMC2814048

[B55] Hotamisligil G. S. (2010). Endoplasmic reticulum stress and the inflammatory basis of metabolic disease. Cell 140, 900–917 10.1016/j.cell.2010.02.034 20303879 PMC2887297

[B56] Hur K. Y. Jung H. S. Lee M. S. (2010). Role of autophagy in beta-cell function and mass. Diabetes Obes. Metab. 12(Suppl. 2), 20–26 10.1111/j.1463-1326.2010.01278.x 21029296

[B57] Imai J. Katagiri H. Yamada T. Ishigaki Y. Suzuki T. Kudo H. (2008). Regulation of pancreatic beta cell mass by neuronal signals from the liver. Science 322, 1250–1254 10.1126/science.1163971 19023081

[B58] Jones H. B. Nugent D. Jenkins R. (2010). Variation in characteristics of islets of Langerhans in insulin-resistant, diabetic and non-diabetic-rat strains. Int. J. Exp. Pathol. 91, 288–301 10.1111/j.1365-2613.2010.00713.x 20384904 PMC2884097

[B59] Kaneto H. Matsuoka T. A. Nakatani Y. Kawamori D. Miyatsuka T. Matsuhisa M. (2005). Oxidative stress, ER stress, and the JNK pathway in type 2 diabetes. J. Mol. Med. (Berl.) 83, 429–439 10.1007/s00109-005-0640-x 15759102

[B60] Karaca M. Magnan C. Kargar C. (2009). Functional pancreatic beta-cell mass: involvement in type 2 diabetes and therapeutic intervention. Diabetes Metab. 35, 77–84 10.1016/S1262-3636(09)72010-5 19251449

[B61] Kasuga M. (2006). Insulin resistance and pancreatic beta cell failure. J. Clin. Invest. 116, 1756–1760 10.1172/JCI29189 16823472 PMC1483164

[B62] Kiraly M. A. Bates H. E. Kaniuk N. A. Yue J. T. Brumell J. H. Matthews S. G. (2008). Swim training prevents hyperglycemia in ZDF rats: mechanisms involved in the partial maintenance of beta-cell function. Am. J. Physiol. Endocrinol. Metab. 294, E271–E283 10.1152/ajpendo.00476.2007 18029442

[B63] Koulmanda M. Bhasin M. Awdeh Z. Qipo A. Fan Z. Hanidziar D. (2012). The role of TNF-alpha in mice with type 1- and 2-diabetes. PLoS ONE 7:e33254 10.1371/journal.pone.0033254 22606220 PMC3350520

[B64] Kulkarni R. N. Jhala U. S. Winnay J. N. Krajewsk S. Montminy M. Kahn C. R. (2004). PDX-1 haploinsufficiency limits the compensatory islet hyperplasia that occurs in response to insulin resistance. J. Clin. Invest. 114, 828–836 10.1172/JCI200421845 15372107 PMC516265

[B65] Kwon G. Marshall C. A. Pappan K. L. Remedi M. S. McDaniel M. L. (2004). Signaling elements involved in the metabolic regulation of mTOR by nutrients, incretins, and growth factors in islets. Diabetes 53(Suppl. 3), S225–S232 10.2337/diabetes.53.4.899 15561916

[B66] Las G. Shirihai O. S. (2010). The role of autophagy in beta-cell lipotoxicity and type 2 diabetes. Diabetes Obes. Metab. 12(Suppl. 2), 15–19 10.1111/j.1463-1326.2010.01268.x 21029295 PMC3786363

[B67] Lau J. Svensson J. Grapensparr L. Johansson A. Carlsson P. O. (2012). Superior beta cell proliferation, function and gene expression in a subpopulation of rat islets identified by high blood perfusion. Diabetologia 55, 1390–1399 10.1007/s00125-012-2476-6 22311418

[B68] LeBras S. Czernichow P. Scharfmann R. (1998). A search for tyrosine kinase receptors expressed in the rat embryonic pancreas. Diabetologia 41, 1474–1481 10.1007/s001250051094 9867215

[B69] Lecompte S. Pasquetti G. Hermant X. Grenier-Boley B. Gonzalez-Gross M. De H. S. (2013). Genetic and molecular insights into the role of PROX1 in glucose metabolism. Diabetes (in press). 10.2337/db12-0864 PMC3636631 23274905

[B70] Lee J. Giordano S. Zhang J. (2012). Autophagy, mitochondria and oxidative stress: cross-talk and redox signalling. Biochem. J. 441, 523–540 10.1042/BJ20111451 22187934 PMC3258656

[B71] Lenzen S. (2008). Oxidative stress: the vulnerable beta-cell. Biochem. Soc. Trans. 36, 343–347 10.1042/BST0360343 18481954

[B72] Li J. Quirt J. Do H. Q. Lyte K. Fellows F. Goodyer C. G. (2007). Expression of c-Kit receptor tyrosine kinase and effect on beta-cell development in the human fetal pancreas. Am. J. Physiol. Endocrinol. Metab. 293, E475–E483 10.1152/ajpendo.00451.2006 17519280

[B73] Lin C. Y. Ni C. C. Yin M. C. Lii C. K. (2012). Flavonoids protect pancreatic beta-cells from cytokines mediated apoptosis through the activation of PI3-kinase pathway. Cytokine 59, 65–71 10.1016/j.cyto.2012.06.060 22579112

[B74] Liu Y. Q. Montanya E. Leahy J. L. (2001). Increased islet DNA synthesis and glucose-derived lipid and amino acid production in association with beta-cell hyperproliferation in normoglycaemic 60% pancreatectomy rats. Diabetologia 44, 1026–1033 10.1007/s001250100597 11484081

[B75] Loder M. K. daSilva Xavier G. McDonald A. Rutter G. A. (2008). TCF7L2 controls insulin gene expression and insulin secretion in mature pancreatic beta-cells. Biochem. Soc. Trans. 36, 357–359 10.1042/BST0360357 18481957

[B76] Lopez-Acosta J. F. Moreno-Amador J. L. Jimenez-Palomares M. Diaz-Marrero A. R. Cueto M. Perdomo G. (2013). Epoxypukalide induces proliferation and protects against cytokine-mediated apoptosis in primary cultures of pancreatic beta-cells. PLoS ONE 8:e52862 10.1371/journal.pone.0052862 23300997 PMC3534672

[B77] Maedler K. Schumann D. M. Schulthess F. Oberholzer J. Bosco D. Berney T. (2006). Aging correlates with decreased beta-cell proliferative capacity and enhanced sensitivity to apoptosis: a potential role for Fas and pancreatic duodenal homeobox-1. Diabetes 55, 2455–2462 10.2337/db05-1586 16936193

[B78] Malin S. K. Kirwan J. P. (2012). Fasting hyperglycaemia blunts the reversal of impaired glucose tolerance after exercise training in obese older adults. Diabetes Obes. Metab. 14, 835–841 10.1111/j.1463-1326.2012.01608.x 22510250 PMC3407343

[B79] Manning A. K. Hivert M. F. Scott R. A. Grimsby J. L. Bouatia-Naji N. Chen H. (2012). A genome-wide approach accounting for body mass index identifies genetic variants influencing fasting glycemic traits and insulin resistance. Nat. Genet. 44, 659–669 10.1038/ng.2274 22581228 PMC3613127

[B80] Marchetti P. Bugliani M. Lupi R. Marselli L. Masini M. Boggi U. (2007). The endoplasmic reticulum in pancreatic beta cells of type 2 diabetes patients. Diabetologia 50, 2486–2494 10.1007/s00125-007-0816-8 17906960

[B81] Marchetti P. Masini M. (2009). Autophagy and the pancreatic beta-cell in human type 2 diabetes. Autophagy 5, 1055–1056 10.4161/auto.5.7.9511 19657235

[B82] Masini M. Bugliani M. Lupi R. Del Guerra S. Boggi U. Filipponi F. (2009). Autophagy in human type 2 diabetes pancreatic beta cells. Diabetologia 52, 1083–1086 10.1007/s00125-009-1347-2 19367387

[B83] McCarthy M. I. (2010). Genomics, type 2 diabetes, and obesity. N. Engl. J. Med. 363, 2339–2350 10.1056/NEJMra0906948 21142536

[B84] McCarthy M. I. Hattersley A. T. (2008). Learning from molecular genetics: novel insights arising from the definition of genes for monogenic and type 2 diabetes. Diabetes 57, 2889–2898 10.2337/db08-0343 18971436 PMC2570381

[B85] Meier J. J. Butler A. E. Saisho Y. Monchamp T. Galasso R. Bhushan A. (2008). Beta-cell replication is the primary mechanism subserving the postnatal expansion of beta-cell mass in humans. Diabetes 57, 1584–1594 10.2337/db07-1369 18334605 PMC3697779

[B86] Mellado-Gil J. M. Cobo-Vuilleumier N. Gauthier B. R. (2012). Islet beta-cell mass preservation and regeneration in diabetes mellitus: four factors with potential therapeutic interest. J. Transplant. 2012, 230870 22919462 10.1155/2012/230870 PMC3420151

[B87] Michael M. D. Kulkarni R. N. Postic C. Previs S. F. Shulman G. I. Magnuson M. A. (2000). Loss of insulin signaling in hepatocytes leads to severe insulin resistance and progressive hepatic dysfunction. Mol. Cell 6, 87–97 10.1016/S1097-2765(00)00010-1 10949030

[B88] Montanya E. Nacher V. Biarnes M. Soler J. (2000). Linear correlation between beta-cell mass and body weight throughout the lifespan in Lewis rats: role of beta-cell hyperplasia and hypertrophy. Diabetes 49, 1341–1346 10.2337/diabetes.49.8.1341 10923635

[B89] Mulder H. Ling C. (2009). Mitochondrial dysfunction in pancreatic beta-cells in type 2 diabetes. Mol. Cell. Endocrinol. 297, 34–40 10.1016/j.mce.2008.05.015 18606489

[B90] Muoio D. M. Newgard C. B. (2008). Mechanisms of disease: molecular and metabolic mechanisms of insulin resistance and beta-cell failure in type 2 diabetes. Nat. Rev. Mol. Cell Biol. 9, 193–205 10.1038/nrm2327 18200017

[B91] Nir T. Melton D. A. Dor Y. (2007). Recovery from diabetes in mice by beta cell regeneration. J. Clin. Invest. 117, 2553–2561 10.1172/JCI32959 17786244 PMC1957545

[B92] Oberg C. Waltenberger J. Claesson-Welsh L. Welsh M. (1994). Expression of protein tyrosine kinases in islet cells: possible role of the Flk-1 receptor for beta-cell maturation from duct cells. Growth Factors 10, 115–126 10.3109/08977199409010985 7520714

[B93] Oberg-Welsh C. Welsh M. (1996). Effects of certain growth factors on in vitro maturation of rat fetal islet-like structures. Pancreas 12, 334–339 10.1097/00006676-199605000-00002 8740398

[B94] Okada T. Liew C. W. Hu J. Hinault C. Michael M. D. Krtzfeldt J. (2007). Insulin receptors in beta-cells are critical for islet compensatory growth response to insulin resistance. Proc. Natl. Acad. Sci. U.S.A. 104, 8977–8982 10.1073/pnas.0608703104 17416680 PMC1885613

[B95] Ovalle F. Bell D. S. (2002). Clinical evidence of thiazolidinedione-induced improvement of pancreatic beta-cell function in patients with type 2 diabetes mellitus. Diabetes Obes. Metab. 4, 56–59 10.1046/j.1463-1326.2002.00183.x 11874443

[B96] Papas K. K. Colton C. K. Nelson R. A. Rozak P. R. Avgoustiniatos E. S. Scott W. E. (2007). Human islet oxygen consumption rate and DNA measurements predict diabetes reversal in nude mice. Am. J. Transplant. 7, 707–713 10.1111/j.1600-6143.2006.01655.x 17229069 PMC2857994

[B97] Paris M. Bernard-Kargar C. Berthault M. F. Bouwens L. Ktorza A. (2003). Specific and combined effects of insulin and glucose on functional pancreatic beta-cell mass in vivo in adult rats. Endocrinology 144, 2717–2727 10.1210/en.2002-221112 12746336

[B98] Park Y. J. Ao Z. Kieffer T. J. Chen H. Safikhan N. Thompson D. M. (2013). The glucagon-like peptide-1 receptor agonist exenatide restores impaired pro-islet amyloid polypeptide processing in cultured human islets: implications in type 2 diabetes and islet transplantation. Diabetologia 56, 508–519 10.1007/s00125-012-2802-z 23262664

[B99] Parsons J. A. Brelje T. C. Sorenson R. L. (1992). Adaptation of islets of Langerhans to pregnancy: increased islet cell proliferation and insulin secretion correlates with the onset of placental lactogen secretion. Endocrinology 130, 1459–1466 10.1210/en.130.3.1459 1537300

[B100] Pelzer T. Jazbutyte V. Arias-Loza P. A. Segerer S. Lichtenwald M. Law M. P. (2005). Pioglitazone reverses down-regulation of cardiac PPARgamma expression in Zucker diabetic fatty rats. Biochem. Biophys. Res. Commun. 329, 726–732 10.1016/j.bbrc.2005.02.029 15737646

[B101] Petersen K. F. Dufour S. Befroy D. Lehrke M. Hendler R. E. Shulman G. I. (2005). Reversal of nonalcoholic hepatic steatosis, hepatic insulin resistance, and hyperglycemia by moderate weight reduction in patients with type 2 diabetes. Diabetes 54, 603–608 10.2337/diabetes.54.3.603 15734833 PMC2995496

[B102] Petrie J. R. Pearson E. R. Sutherland C. (2011). Implications of genome wide association studies for the understanding of type 2 diabetes pathophysiology. Biochem. Pharmacol. 81, 471–477 10.1016/j.bcp.2010.11.010 21111713

[B103] Petrik J. Arany E. McDonald T. J. Hill D. J. (1998). Apoptosis in the pancreatic islet cells of the neonatal rat is associated with a reduced expression of insulin-like growth factor II that may act as a survival factor. Endocrinology 139, 2994–3004 10.1210/en.139.6.2994 9607811

[B104] Pinnick K. E. Collins S. C. Londos C. Gauguier D. Clark A. Fielding B. A. (2008). Pancreatic ectopic fat is characterized by adipocyte infiltration and altered lipid composition. Obesity (Silver Spring) 16, 522–530 10.1038/oby.2007.110 18239594

[B105] Poitout V. Robertson R. P. (2008). Glucolipotoxicity: fuel excess and beta-cell dysfunction. Endocr. Rev. 29, 351–366 10.1210/er.2007-0023 18048763 PMC2528858

[B106] Porat S. Weinberg-Corem N. Tornovsky-Babaey S. Schyr-Ben-Haroush R. Hija A. Stolovich-Rain M. (2011). Control of pancreatic beta cell regeneration by glucose metabolism. Cell Metab. 13, 440–449 10.1016/j.cmet.2011.02.012 21459328 PMC11807376

[B107] Prudente S. Morini E. Trischitta V. (2009). Insulin signaling regulating genes: effect on T2DM and cardiovascular risk. Nat. Rev. Endocrinol. 5, 682–693 10.1038/nrendo.2009.215 19924153

[B108] Rachdi L. El G. L. Bernex F. Panthier J. J. Czernichow P. Scharfmann R. (2001). Expression of the receptor tyrosine kinase KIT in mature beta-cells and in the pancreas in development. Diabetes 50, 2021–2028 10.2337/diabetes.50.9.2021 11522667

[B109] Rahier J. Guiot Y. Goebbels R. M. Sempoux C. Henquin J. C. (2008). Pancreatic beta-cell mass in European subjects with type 2 diabetes. Diabetes Obes. Metab. 10(Suppl. 4), 32–42 10.1111/j.1463-1326.2008.00969.x 18834431

[B110] Rankin M. M. Kushner J. A. (2009). Adaptive beta-cell proliferation is severely restricted with advanced age. Diabetes 58, 1365–1372 10.2337/db08-1198 19265026 PMC2682671

[B111] Rao J. R. Keating D. J. Chen C. Parkington H. C. (2012). Adiponectin increases insulin content and cell proliferation in MIN6 cells via PPARgamma-dependent and PPARgamma-independent mechanisms. Diabetes Obes. Metab. 14, 983–989 10.1111/j.1463-1326.2012.01626.x 22594400

[B112] Reers C. Erbel S. Esposito I. Schmied B. Buchler M. W. Nawroth P. P. (2009). Impaired islet turnover in human donor pancreata with aging. Eur. J. Endocrinol. 160, 185–191 10.1530/EJE-08-0596 19004984

[B113] Reusens B. Theys N. Remacle C. (2011). Alteration of mitochondrial function in adult rat offspring of malnourished dams. World J. Diabetes 2, 149–157 10.4239/wjd.v2.i9.149 21954419 PMC3180527

[B114] Reynoso R. Salgado L. M. Calderon V. (2003). High levels of palmitic acid lead to insulin resistance due to changes in the level of phosphorylation of the insulin receptor and insulin receptor substrate-1. Mol. Cell. Biochem. 246, 155–162 10.1023/A:1023423005187 12841357

[B115] Robertson R. Zhou H. Zhang T. Harmon J. S. (2007). Chronic oxidative stress as a mechanism for glucose toxicity of the beta cell in type 2 diabetes. Cell Biochem. Biophys. 48, 139–146 10.1007/s12013-007-0026-5 17709883

[B116] Robertson R. P. (2004). Chronic oxidative stress as a central mechanism for glucose toxicity in pancreatic islet beta cells in diabetes. J. Biol. Chem. 279, 42351–42354 10.1074/jbc.R400019200 15258147

[B117] Robson-Doucette C. A. Sultan S. Allister E. M. Wikstrom J. D. Koshkin V. Bhattacharjee A. (2011). Beta-cell uncoupling protein 2 regulates reactive oxygen species production, which influences both insulin and glucagon secretion. Diabetes 60, 2710–2719 10.2337/db11-0132 21984579 PMC3198081

[B118] Ryu S. Kodama S. Ryu K. Schoenfeld D. A. Faustman D. L. (2001). Reversal of established autoimmune diabetes by restoration of endogenous beta cell function. J. Clin. Invest. 108, 63–72 10.1172/JCI12335 11435458 PMC209340

[B119] Sachdeva M. M. Claiborn K. C. Khoo C. Yang J. Groff D. N. Mirmira R. G. (2009). Pdx1 (MODY4) regulates pancreatic beta cell susceptibility to ER stress. Proc. Natl. Acad. Sci. U.S.A. 106, 19090–19095 10.1073/pnas.0808042106 19855005 PMC2776433

[B120] Samuel V. T. Shulman G. I. (2012). Mechanisms for insulin resistance: common threads and missing links. Cell 148, 852–871 10.1016/j.cell.2012.02.017 22385956 PMC3294420

[B121] Sarkar A. Zhang M. Liu S. H. Sarkar S. Brunicardi F. C. Berger D. H. (2011). Serum response factor expression is enriched in pancreatic beta cells and regulates insulin gene expression. FASEB J. 25, 2592–2603 10.1096/fj.10-173757 21525490

[B122] Sato Y. Hatta M. Karim M. F. Sawa T. Wei F. Y. Sato S. (2012). Anks4b, a novel target of HNF4alpha interacts with GRP78 and regulates endoplasmic reticulum stress-induced apoptosis in pancreatic beta-cells. J. Biol. Chem. 287, 23236–23245 10.1074/jbc.M111.318311 22589549 PMC3391104

[B123] Scaglia L. Cahill C. J. Finegood D. T. Bonner-Weir S. (1997). Apoptosis participates in the remodeling of the endocrine pancreas in the neonatal rat. Endocrinology 138, 1736–1741 10.1210/en.138.4.1736 9075738

[B124] Scaglia L. Smith F. E. Bonner-Weir S. (1995). Apoptosis contributes to the involution of beta cell mass in the post partum rat pancreas. Endocrinology 136, 5461–5468 10.1210/en.136.12.5461 7588296

[B125] Scherz-Shouval R. Elazar Z. (2011). Regulation of autophagy by ROS: physiology and pathology. Trends Biochem. Sci. 36, 30–38 10.1016/j.tibs.2010.07.007 20728362

[B126] Schrimpe-Rutledge A. C. Fontes G. Gritsenko M. Norbeck A. D. Anderson D. J. Waters K. (2012). Discovery of novel glucose-regulated proteins in isolated human pancreatic islets using LC-MS/MS-based proteomics. J. Proteome Res. 11, 3520–3532 10.1021/pr3002996 22578083 PMC3391329

[B127] Schuit F. Flamez D. De Vos A. Pipeleers D. (2002). Glucose-regulated gene expression maintaining the glucose-responsive state of beta-cells. Diabetes 51(Suppl. 3), 326–332 10.2337/diabetes.51.2007.S326 12475771

[B128] Shimabukuro M. Zhou Y. T. Levi M. Unger R. H. (1998). Fatty acid-induced beta cell apoptosis: a link between obesity and diabetes. Proc. Natl. Acad. Sci. U.S.A. 95, 2498–2502 10.1073/pnas.95.5.2498 9482914 PMC19389

[B129] Shoelson S. E. Lee J. Goldfine A. B. (2006). Inflammation and insulin resistance. J. Clin. Invest. 116, 1793–1801 10.1172/JCI29069 16823477 PMC1483173

[B130] Shu L. Matveyenko A. V. Kerr-Conte J. Cho J. H. McIntosh C. H. Maedler K. (2009). Decreased TCF7L2 protein levels in type 2 diabetes mellitus correlate with downregulation of GIP- and GLP-1 receptors and impaired beta-cell function. Hum. Mol. Genet. 18, 2388–2399 10.1093/hmg/ddp178 19386626 PMC2722186

[B131] Shu L. Sauter N. S. Schulthess F. T. Matveyenko A. V. Oberholzer J. Maedler K. (2008). Transcription factor 7-like 2 regulates beta-cell survival and function in human pancreatic islets. Diabetes 57, 645–653 10.2337/db07-0847 18071026

[B132] Simmons R. A. (2007). Role of metabolic programming in the pathogenesis of beta-cell failure in postnatal life. Rev. Endocr. Metab. Disord. 8, 95–104 10.1007/s11154-007-9045-1 17680370

[B133] Siri-Tarino P. W. Sun Q. Hu F. B. Krauss R. M. (2010). Saturated fat, carbohydrate, and cardiovascular disease. Am. J. Clin. Nutr. 91, 502–509 10.3945/ajcn.2009.27725 20089734 PMC2824150

[B134] Solomon T. P. Haus J. M. Kelly K. R. Rocco M. Kashyap S. R. Kirwan J. P. (2010). Improved pancreatic beta-cell function in type 2 diabetic patients after lifestyle-induced weight loss is related to glucose-dependent insulinotropic polypeptide. Diabetes Care 33, 1561–1566 10.2337/dc09-2021 20200305 PMC2890359

[B135] Steer S. A. Scarim A. L. Chambers K. T. Corbett J. A. (2006). Interleukin-1 stimulates beta-cell necrosis and release of the immunological adjuvant HMGB1. PLoS Med. 3:e17 10.1371/journal.pmed.0030017 16354107 PMC1316065

[B136] Stefanovski D. Richey J. M. Woolcott O. Lottati M. Zheng D. Harrison L. N. (2011). Consistency of the disposition index in the face of diet induced insulin resistance: potential role of FFA. PLoS ONE 6:e18134 10.1371/journal.pone.0018134 21479217 PMC3068147

[B137] Stienstra R. Duval C. Muller M. Kersten S. (2007). PPARs, obesity, and inflammation. PPAR Res. 2007, 95974 10.1155/2007/95974 17389767 PMC1783744

[B138] Stoffers D. A. (2004). The development of beta-cell mass: recent progress and potential role of GLP-1. Horm. Metab. Res. 36, 811–821 10.1055/s-2004-826168 15655713

[B139] Sweet I. R. Gilbert M. Jensen R. Sabek O. Fraga D. W. Gaber A. O. (2005). Glucose stimulation of cytochrome C reduction and oxygen consumption as assessment of human islet quality. Transplantation 80, 1003–1011 10.1097/01.tp.0000178381.35014.37 16278578

[B140] Sweet I. R. Gilbert M. Scott S. Todorov I. Jensen R. Nair I. (2008). Glucose-stimulated increment in oxygen consumption rate as a standardized test of human islet quality. Am. J. Transplant. 8, 183–192 18021279 10.1111/j.1600-6143.2007.02041.x

[B141] Talchai C. Xuan S. Lin H. V. Sussel L. Accili D. (2012). Pancreatic beta cell dedifferentiation as a mechanism of diabetic beta cell failure. Cell 150, 1223–1234 10.1016/j.cell.2012.07.029 22980982 PMC3445031

[B142] Tarabra E. Pelengaris S. Khan M. (2012). A simple matter of life and death-the trials of postnatal beta-cell mass regulation. Int. J. Endocrinol. 2012, 516718 22577380 10.1155/2012/516718 PMC3346985

[B143] Teta M. Long S. Y. Wartschow L. M. Rankin M. M. Kushner J. A. (2005). Very slow turnover of beta-cells in aged adult mice. Diabetes 54, 2557–2567 10.2337/diabetes.54.9.2557 16123343

[B144] Teta M. Rankin M. M. Long S. Y. Stein G. M. Kushner J. A. (2007). Growth and regeneration of adult beta cells does not involve specialized progenitors. Dev. Cell 12, 817–826 10.1016/j.devcel.2007.04.011 17488631

[B145] Trujillo M. E. Scherer P. E. (2006). Adipose tissue-derived factors: impact on health and disease. Endocr. Rev. 27, 762–778 17056740 10.1210/er.2006-0033

[B146] Tsuneoka M. Teye K. Arima N. Soejima M. Otera H. Ohashi K. (2005). A novel Myc-target gene, mimitin, that is involved in cell proliferation of esophageal squamous cell carcinoma. J. Biol. Chem. 280, 19977–19985 10.1074/jbc.M501231200 15774466

[B147] Tushuizen M. E. Bunck M. C. Pouwels P. J. Bontemps S. van Waesberghe J. H. Schindhelm R. K. (2007). Pancreatic fat content and beta-cell function in men with and without type 2 diabetes. Diabetes Care 30, 2916–2921 10.2337/dc06-1473 17666465

[B148] Van Assche F. A. Aerts L. De P. F. (1978). A morphological study of the endocrine pancreas in human pregnancy. Br. J. Obstet. Gynaecol. 85, 818–820 10.1111/j.1471-0528.1978.tb15835.x 363135

[B149] Vessby B. Tengblad S. Lithell H. (1994). Insulin sensitivity is related to the fatty acid composition of serum lipids and skeletal muscle phospholipids in 70-year-old men. Diabetologia 37, 1044–1050 10.1007/BF00400468 7851683

[B150] Voight B. F. Scott L. J. Steinthorsdottir V. Morris A. P. Dina C. Welch R. P. (2010). Twelve type 2 diabetes susceptibility loci identified through large-scale association analysis. Nat. Genet. 42, 579–589 10.1038/ng.609 20581827 PMC3080658

[B151] Wang S. Hecksher-Sorensen J. Xu Y. Zhao A. Dor Y. Rosenberg L. (2008). Myt1 and Ngn3 form a feed-forward expression loop to promote endocrine islet cell differentiation. Dev. Biol. 317, 531–540 10.1016/j.ydbio.2008.02.052 18394599 PMC2423199

[B152] Weickert M. O. (2012). What dietary modification best improves insulin sensitivity and why? Clin. Endocrinol. (Oxf.) 77, 508–512 10.1111/j.1365-2265.2012.04450.x 22640465

[B153] Weir G. C. Laybutt D. R. Kaneto H. Bonner-Weir S. Sharma A. (2001). Beta-cell adaptation and decompensation during the progression of diabetes. Diabetes 50(Suppl. 1), 154–159 10.2337/diabetes.50.2007.S154 11272180

[B154] Welsh M. Anneren C. Lindholm C. Kriz V. Oberg-Welsh C. (2000). Role of tyrosine kinase signaling for beta-cell replication and survival. Ups. J. Med. Sci. 105, 7–15 11095102 10.1517/03009734000000052

[B155] Wikstrom J. D. Sereda S. B. Stiles L. Elorza A. Allister E. M. Neilson A. (2012). A novel high-throughput assay for islet respiration reveals uncoupling of rodent and human islets. PLoS ONE 7:e33023 10.1371/journal.pone.0033023 22606219 PMC3351473

[B156] Wu J. Wu J. J. Yang L. J. Wei L. X. Zou D. J. (2013). Rosiglitazone protects against palmitate-induced pancreatic beta-cell death by activation of autophagy via 5’-AMP-activated protein kinase modulation. Endocrine (in press). 10.1007/s12020-012-9826-5 23109223

[B157] Yaney G. C. Corkey B. E. (2003). Fatty acid metabolism and insulin secretion in pancreatic beta cells. Diabetologia 46, 1297–1312 10.1007/s00125-003-1207-4 13680127

[B158] Yashpal N. K. Li J. Wang R. (2004). Characterization of c-Kit and nestin expression during islet cell development in the prenatal and postnatal rat pancreas. Dev. Dyn. 229, 813–825 10.1002/dvdy.10496 15042705

[B159] Yuzefovych L. V. Musiyenko S. I. Wilson G. L. Rachek L. I. (2013). Mitochondrial DNA damage and dysfunction, and oxidative stress are associated with endoplasmic reticulum stress, protein degradation and apoptosis in high fat diet-induced insulin resistance mice. PLoS ONE 8:e54059 10.1371/journal.pone.0054059 23342074 PMC3546973

[B160] Zhang Y. Zhang Y. Bone R. N. Cui W. Peng J. B. Siegal G. P. (2012). Regeneration of pancreatic non-beta endocrine cells in adult mice following a single diabetes-inducing dose of streptozotocin. PLoS ONE 7:e36675 10.1371/journal.pone.0036675 22586489 PMC3346729

